# Application of Artificial Neural Networks for Prediction of Received Signal Strength Indication and Signal-to-Noise Ratio in Amazonian Wooded Environments

**DOI:** 10.3390/s24082542

**Published:** 2024-04-16

**Authors:** Brenda S. de S. Barbosa, Hugo A. O. Cruz, Alex S. Macedo, Caio M. M. Cardoso, Filipe C. Fernandes, Leslye E. C. Eras, Jasmine P. L. de Araújo, Gervásio P. S. Calvacante, Fabrício J. B. Barros

**Affiliations:** 1 Electrical Engineering Graduate Department, Universidade Federal do Pará, Rua Augusto Corrêa, 01, Guamá, Belém 66075-110, Brazil; hugo1822@gmail.com (H.A.O.C.); alexsanchess@gmail.com (A.S.M.); caio.cardoso@itec.ufpa.br (C.M.M.C.); filipe.fernandes@itec.ufpa.br (F.C.F.); jasmine@ufpa.br (J.P.L.d.A.); gervasio@ufpa.br (G.P.S.C.); fbarros@ufpa.br (F.J.B.B.); 2 Institute of Geoscience and Engineering, Universidade Federal do Sul e Sudoeste do Pará, Marabá 68505-080, Brazil; lecastro@unifesspa.edu.br

**Keywords:** Amazon, dense vegetation, LoRa, machine learning, propagation models

## Abstract

The presence of green areas in urbanized cities is crucial to reduce the negative impacts of urbanization. However, these areas can influence the signal quality of IoT devices that use wireless communication, such as LoRa technology. Vegetation attenuates electromagnetic waves, interfering with the data transmission between IoT devices, resulting in the need for signal propagation modeling, which considers the effect of vegetation on its propagation. In this context, this research was conducted at the Federal University of Pará, using measurements in a wooded environment composed of the Pau-Mulato species, typical of the Amazon. Two machine learning-based propagation models, GRNN and MLPNN, were developed to consider the effect of Amazonian trees on propagation, analyzing different factors, such as the transmitter’s height relative to the trunk, the beginning of foliage, and the middle of the tree canopy, as well as the LoRa spreading factor (SF) 12, and the co-polarization of the transmitter and receiver antennas. The proposed models demonstrated higher accuracy, achieving values of root mean square error (RMSE) of 3.86 dB and standard deviation (SD) of 3.8614 dB, respectively, compared to existing empirical models like CI, FI, Early ITU-R, COST235, Weissberger, and FITU-R. The significance of this study lies in its potential to boost wireless communications in wooded environments. Furthermore, this research contributes to enhancing more efficient and robust LoRa networks for applications in agriculture, environmental monitoring, and smart urban infrastructure.

## 1. Introduction

The Internet of Things (IoT) is a reality that impacts all the telecommunication sectors, contributing to the evolution of numerous applications, such as smart cities, smart campuses, and smart agriculture. The connected things gather information that helps improve every type of process, saving financial resources and energy. Furthermore, the IoT market is expected to reach USD 1.5 trillion by 2030 [1].

In summary, the Internet of Things is an idea that has already impacted and will continue to impact all aspects of human life significantly [2]. Moreover, most devices used in IoT will employ non-cellular wireless technologies, such as Bluetooth^®^, Wi-Fi, ZigBee, THREAD, EnOcean, Sigfox^®^, and LoRa™, among others [3].

IoT devices have different bandwidth, rate, and transmission power requirements. Additionally, they are subject to interference from other wireless transmitters, path loss, random fluctuations caused by shadowing, and obstacles in the communication path [4].

These factors cause high variability in the quality of the received signal, affecting the reliability and performance of wireless communication systems. Therefore, it is necessary to carry out studies to guarantee the reliability and coverage of the connection, making it essential to develop propagation loss models for the most diverse environments [4].

Several studies have addressed the modeling of communication channels, considering different wireless communication technologies. Among these technologies, Long-Range (LoRa) has stood out, as it provides long-range communication and low energy consumption, as pointed out by [5,6]. Moreover, a single receiver in the LoRa network can manage multiple nodes distributed in the environment, leading to significant improvements in IoT applications and contributing to reduce the costs associated with IoT systems deployment.

In addition, channel modeling also varies according to the characteristics of the surrounding area. The combination of dense vegetation, topographical variations, high humidity, remote locations, and wildlife interference makes the Amazon rainforest a particularly challenging environment for wireless signal propagation. This environmental diversity represents a challenge for planning systems to design and deploy IoT applications efficiently. Without understanding the appropriate channel model, it is difficult to analyze coverage and predict optimal deployment configurations.

Empirical propagation loss models in different outdoor environments have been proposed in [7,8,9], acknowledging that the type of terrain significantly interferes with signal propagation and the importance of accurate models to assist in decision-making for the wireless sensor networks’ (WSN) deployment. Therefore, outdoor environments with heterogeneous vegetation have the special characteristic of acting as spreaders of electromagnetic waves. The excessive spread attenuates the signal, limiting the performance expected for 5G mobile wireless communications [10].

Works such as [11,12,13] mentioning the use of machine learning (ML) techniques to predict propagation loss has been progressively growing. The use of ML techniques to model the wireless signal channel improves the propagation loss prediction in different environments. However, to use ML models, it is necessary to realize data acquisition, which will be employed to train different models. In addition, to the best of the authors’ acknowledge, few are the works that collect data to represent signal propagation in an Amazon wooded environment, as seen in [14,15,16,17,18,19].

In this context, this research aims to develop an ML model that represents the channel behavior in an Amazon wooded environment. For this purpose, a measurement campaign was conducted in the Camilo Vianna forest, located within the Federal University of Pará (UFPA). Furthermore, the developed ML model was compared with classical models present in the literature to verify the model quality.

Thus, the main contributions of this research are as follows:

- Extensive measurements in the Amazon environment with the transmitter at different heights: 6 m (tree trunk level), 12 m (beginning of the tree canopy), and 18 m (middle of the tree canopy);
- Analysis of signal propagation in the Amazon environment considering different polarizations of the transmitting and receiving antennas, vertical–vertical (VV) and horizontal–horizontal (HH);
- Analysis of the RSSI (received signal strength indication) and SNR (signal-to-noise ratio) variation for Amazonian environments;
- Calculation of coefficients, Alpha and Beta, for the Floating Intercept (FI) model and Path Loss Exponent (PLE) for the Close-In (CI) considering the frequency of 915 MHz for environments with heterogeneous and dense vegetation;
- Development of two ML models using a general regression neural network (GRNN) and a multilayer perceptron neural network (MLPNN), based on data measured in a densely wooded scenario.

The rest of this article is organized as follows. Section 2 discusses with details the related works showing the main works with propagation in wooded environments and addresses ML-based propagation models. Section 3 describes the materials and methods used in this research. Section 4 presents the main empirical propagation models, used to compare with the proposed model. Section 5 explains the machine learning techniques employed in this work. Section 6 details the evaluation of the results found by this work. Finally, Section 7 provides the conclusions of the research and suggest future works.

## 2. Related Works

The performance of LoRa technology in both indoor and outdoor environments has been the subject of various research studies. However, there is a limited number of articles specifically addressing LoRa in vegetated environments. The authors in [20] explore LoRa performance in a unique environment, namely the ISTAC-IIUM Campus, featuring a row of five palm trees. Utilizing different spreading factors (SF7-SF12) at 868 MHz, 125 KHz bandwidth, and a transmission power of 14 dBm, the study measures the received signal strength indication (RSSI). It highlights factors impacting LoRa propagation through vegetation, including diffraction, foliage scattering, and reflection, emphasizing the importance of considering these aspects to optimize LoRa performance in vegetated areas and overcome associated challenges.

The article [21] presents a study about LPWAN for smart agriculture applications, evaluating the performance of LoRa transmission technology operating in the 433 MHz and 868 MHz bands, intended for wildlife monitoring in a forested vegetation area. The study analyzed signal-to-noise ratio (SNR), RSSI, and packet delivery rate (PDR), showing that the stability of the LoRa signal significantly depends on the environment and is more stable in less dense forest environments than in highly dense forest environments. Furthermore, it suggests future research regarding the study of environmental impact, considering atmospheric conditions (humidity, pressure, rainfall, etc.), LoRa performance, and proposing a suitable propagation model for forested environments based on the obtained results.

Aiming to obtain a suitable propagation model for vegetated environments, the article [22] investigates the use of empirical propagation models in precision agriculture. Models such as the Free Space Path Loss (FSPL) for large-scale loss in free space, the Flat Earth (FE) model, and vegetation models like Weissberger, ITU-R, FITU-R, and COST235 are explored in a simulation, assuming flat terrain greenhouse with an area of 100 m × 100 m and operation frequency of 2.5 GHz. The study considers variations in antenna heights, distance between transceivers, and vegetation depth to predict signal propagation loss. At the end of the simulation, the study explains that propagation losses decrease with great antenna heights and increase with large distances between transceivers and deeper vegetation. The total loss is calculated as the sum of free space loss and vegetation loss in greenhouse-style environments.

The article [23] proposes a method to improve the accuracy of path loss for wireless communication in a mountainous forest area called Takakuma at Kagoshima University, Tarumizu City, southern Japan, at an elevation of 2000 m. The devices operate at a frequency of 920 MHz, SF12, transmission power of 13 dBm, a bandwidth of 400 KHz, and monopole antennas with vertical polarization at heights of 2 m and 2.5 m for the receiver and transmitter, respectively. This method divides the total distance between the transmitter and receiver into two parts: one is the total transmission distance through the forest, and the other is the total distance considering free space. Subsequently, it calculates the path losses for the forested area and the open space, and then sums them to obtain the total path loss. Finally, the study compares the proposed method with five literature models Weissberger, ITU-R, FITU-R, LITU-R, and COST235, and concludes that the proposed method exhibits a reasonable level of path loss accuracy in mountainous forest environments and has the potential to enhance the accuracy of wireless communication in such environments.

In [24], the behavior of wireless channels at frequencies of 433 MHz and 2.4 GHz is investigated, considering the effects of distance and antenna height on signal strength and packet loss rate in agricultural areas. The study compares modified models of exponential decay for vegetation environments, including Weissberger, ITU-R, and COST235, along with a linear logarithmic model, through Matlab simulations. An ideal fitting model, namely the parametric exponential decay model (OFPED), is obtained. Measurements are conducted in a wheat field using Zigbee technology, with a transmission power of 0 dBm and a 4 dB gain omnidirectional antenna. RSSI and Packet Loss Rate (PLR) are obtained at different distances from the transceivers and various heights of the transmitter and receiver antennas, considering three stages of wheat growth. The findings reveal that the RSSI decreases as the distance from the transceiver increases, and the PLR increases with the distance from the transceiver. Additionally, path loss decreases with increasing antenna height, and the path loss at 2.4 GHz is higher than that at 433 MHz. Finally, the validation results indicate that modified exponential decay (MED) models can be used as conservative upper and lower limits for path loss, at least for the wheat field.

In [25], the study focuses on deploying WSN in high grass environments. Received signal strength (RSS) measurements were collected on an 80 m × 80 m high grass farm, where the transmitter and receiver were always in line-of-sight (LOS). The transmission antenna was placed at the center of the grassy area, and the receiving antennas were positioned at eight different distances. These antennas’ height was 20 cm, with omnidirectional radiation patterns. Data collection was carried out at a frequency of 1925 MHz, at a total of 128 measurement points, with 300 RSS samples collected at each point. As a result, it was possible to propose an empirical propagation loss model for this type of scenario, which was compared to classical models such as FSPL and Two-Ray Ground, both of which were less accurate in predicting RSS in high grass environments.

The article [26] provides a systematic literature review of propagation models for WSN in vegetated environments, including references to articles such as [20,22,23,24,25]. The review indicates that, from 2011 to 2021, Zigbee technology was preferred for such environments, but currently, the use of LoRa technology is on the rise. Furthermore, the article suggests employing techniques not previously used in wireless communication propagation studies in these scenarios, such as machine learning.

In addition, the article [27] provides an overview of recent developments in radio wave propagation modeling using ML algorithms. It emphasizes the importance of selecting the appropriate ML method for the propagation model accuracy and notes the significant number of propagation modeling articles that have utilized artificial neural networks (ANNs). The article anticipates a further growth in this trend, highlighting the challenges, prospects, and open issues in this research direction.

Some related articles cited, such as [28], employ the back-propagation (BP) neural network to predict the received signal power in a suburban environment. This model is based on field measurements obtained from the base station (BS) and the receiver (Rx), including information on topography, frequency, transmitted power, antenna angle, and received power at all locations. The results demonstrate that the proposed model accurately predicts the received signal power for this type of environment.

The article [29] uses a combination of three techniques: multidimensional regression based on ANN, analysis of variance based on Gaussian process, and feature selection aided by principal component analysis (PCA), and path loss data measured in a suburban area in Korea are used. The collected data show that the proposed combined path loss and shading model is more accurate and flexible compared to the conventional log-normal path loss model. The article [11] presents a study on the prediction of digital terrestrial television (DTT) coverage using ML regression techniques, through electric field intensity measurements in eight DTT channels operating in the city of Quito in Ecuador, showing the efficiency of using ML in this application.

Furthermore, the article [30] conducted measurements in Cyprus considering six transceiver BSs, at frequencies of 433 MHz and 2.4 GHz. The mobile devices used in these field measurements were Sony Z2 phones equipped with TEMS for measuring received power and other key performance indicators (KPIs) of the investigated network. The mobile device’s height was 1.5 m, with an average propagation distance of around 3.0 km and a specified measurement time of about 60 s on average. The aim was to investigate the impact of the plants’ height and antenna height, at various stages of plants growth, on signal propagation through RSSI and PLR analysis. From these data, two ML models are proposed: the radial basis function neural network (RBFNN) and the multilayer perception neural network (MLPNN), compared with the following empirical models: free space, COST231 Hata, ECC-33, Ericsson, and ITU-R, through the metrics RMSE, MAPE, MAE, and $R^{2}$, showing that RBFNN performed better than MLPNN and predicts the loss propagation closest to the measured data with minimal error.

As can be seen in the works cited above, none of them addressed the analysis of the transmission and reception antennas matching polarizations. Moreover, the works do not analyze the different heights of the transmitter in environments with dense vegetation, relating the transmitter height to different parts of the trees. Furthermore, although some already employ machine learning techniques, none of them use a general regression neural network (GRNN).

Therefore, from this overview of related works, it is noted that this research uses an trending approach, as it presents two neural network models, GRNN and MLPNN, using LoRa technology at the frequency of 915 MHz and considering the co-polarization of antennas, both transmitter and receiver, in Amazonian forest environments, to predict RSSI and SNR with greater accuracy.

## 3. Materials and Methods

**Figure 1:**
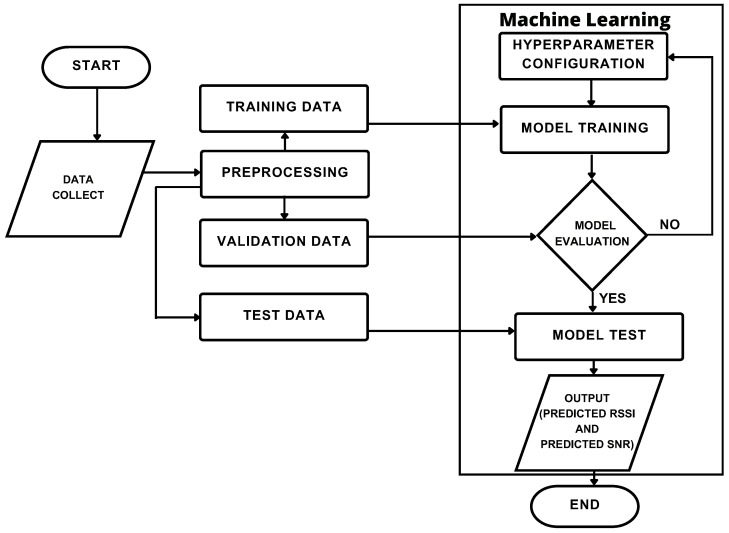
Flowchart of the applied methodology.

The methodology applied in this research is divided into the following stages: data collection (RSSI, SNR, and geolocations) related to signal transmission in an area of dense vegetation, followed by data pre-processing to handle and apply the proposed ML techniques for RSSI and SNR prediction. Subsequently, propagation loss is calculated according to the flowchart in Figure 1.

### 3.1. Description of the Measurement Campaign

This section will present the methodology applied for data collection of the wireless signal in a wooded scenario, describing the devices and configurations for transmitting the LoRa signal. This will take into account the distance between Tx-Rx, Tx height, and detailing the pre-processing steps of the data obtained.

#### 3.1.1. Scenario

**Figure 2:**
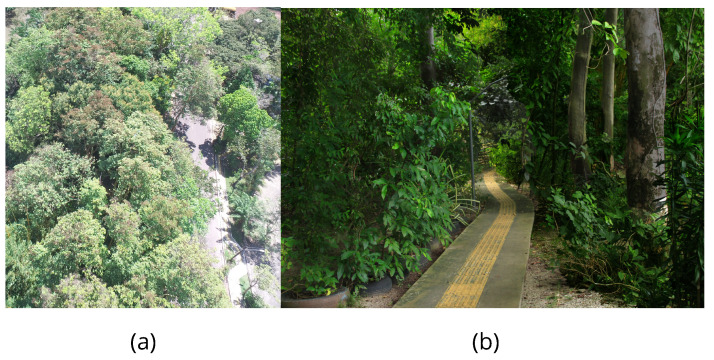
Measurement scenario Camilo Vianna forest (UFPA): (**a**) Aerial view of the Amazon rainforest grove. (**b**) Path of the tree-lined corridor traversed for data collection within the grove.

The measurements were conducted in Belém, Pará, at the campus of the Federal University of Pará (UFPA). The University City contains small forests formed by native species with tree, shrub, and forage vegetation. Among these forests, the Camillo Vianna forest or Pau-Mulato forest, which occupies an area of 16.700 $m^{2}$, was chosen due to the presence of several specimens of Pau-Mulato trees, a typical Amazonian tree [31], as shown in Figure 2.

#### 3.1.2. Equipment Setup and Configuration

**Figure 3:**
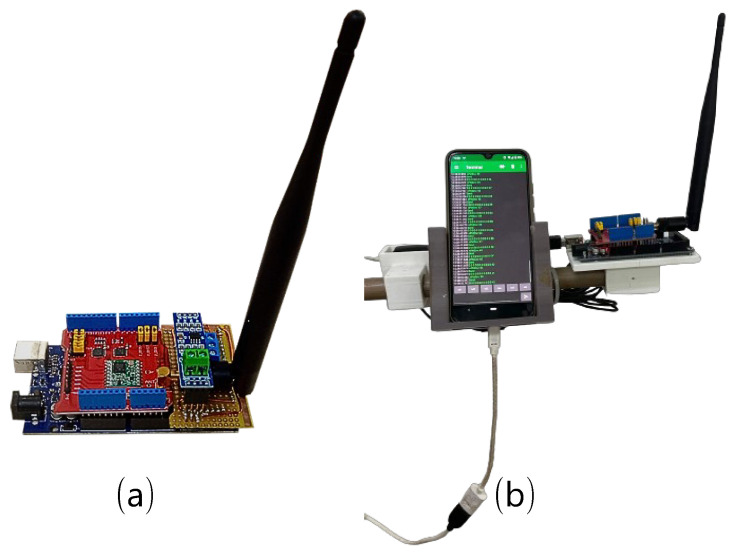
Transmission and reception equipment: (**a**) Arduino Uno module + Dragino 915 MHz LoRa module + omnidirectional antenna. (**b**) Arduino Uno module + Dragino 915 MHz LoRa module + omnidirectional antenna + GPS + smartphone.

For the data collection, three sets of LoRa Dragino shields were used. The shields were coupled to an arduino and used the chip sx1276 from SEMTECH and monopole and omnidirectional antennas [32]. At transmission, a 9 V battery is used, and at reception two smartphones are used to power the circuit and record the transmission log. Table 1 shows the parameters used in measurements and Figure 3 shows the equipment.

The equipment settings shown in Table 1 were chosen for the following reason. The lowest feasible transmit power of 5 dBm was chosen to preserve the device energy, allowing for more measurements to be taken before battery recharging. Moreover, this approach minimizes potential interference with the animals’ natural habitat and aligns with our sustainability goals.

In addition, the bandwidth of 125 kHz and the coding rate of 4/5 were selected because they are the standard for LoRa devices in the AUS915-928 region [33]. Moreover, the 915 MHz frequency was chosen for our study because it is part of the ISM (Industrial, Scientific, and Medical) band allocated for such unlicensed communication purposes in Brazil.

Finally, the SF determines the transmission rate and the sensitivity of the LoRa receiver. For densely wooded environments, SF12 is an ideal option for several important reasons. Firstly, trees and other types of dense vegetation act as physical obstacles that can weaken and attenuate the radio signal. A high SF offers a greater communication range. In addition, the high SF spreads the LoRa signal over a wider bandwidth, which increases the signal’s resilience and extends the transmission range [34].

#### 3.1.3. Measurement Methodology

**Figure 4:**
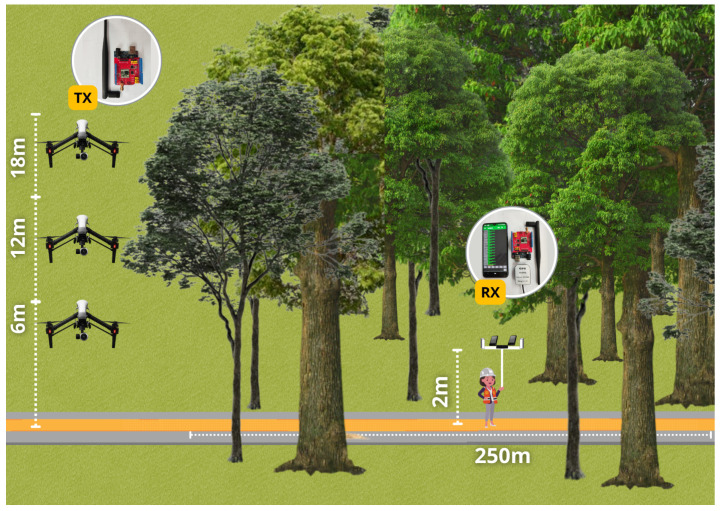
Measurement methodology scenario.

The measurements were based on the transmission and reception of the signal, with the transmitter mounted on a DJI Inspire 1 drone located outside the forest at different heights (6 m, 12 m, and 18 m) and the receivers mounted on a pole with a height of 2 m, being moved by a person walking inside the forest. Both transmitter and receivers had antennas in both vertical and horizontal polarizations, as illustrated in Figure 4.

Considering the morphology of the forest, predominantly composed of the Pau-Mulato tree species, a typical tree of the Amazon [35] has an average height of 24 m. The transmitter heights were defined at 6 m (tree trunk level), 12 m (beginning of the tree canopy), and 18 m (middle of the tree canopy). The aim was to analyze the influence of transmitter heights and antenna polarizations in the forest. Table 2 shows all possible combinations of transmission and reception parameters.

Given that there are one SF, three heights, and two polarizations in a non-line-of-sight (NLOS) scenario, six combinations were tested. For each combination, the 250 m route inside the forest was traveled six times, totaling 36 repetitions of the same route. Approximately 4.525 samples were collected in total, averaging about 358 samples per route, which corresponds to approximately one sample every 2 m while walking at an average speed of approximately 1.67 m/s. Each sample comprised latitude and longitude values, RSSI, and SNR.

### 3.2. Data Processing

Data processing consists of a set of techniques and steps applied to improve, organize, clean, and transform raw data to be analyzed. In addition, it helps to identify and correct errors, noise, and missing values in the data. Furthermore, data processing also addresses the integration and combination of data from different sources, allowing one to create a unified and coherent dataset.

In this research, to carry out data processing, the rule 3-sigma (3−σ) was used to identify and remove outliers. This rule uses three times the standard deviation value from the mean (μ) in a window of 25 samples, sliding this window through the series of measurements. The values μ + (3·σ) and μ−(3·σ) define the upper and lower limits of outliers detection [36].

#### 3.2.1. Distance Calculation

The distance calculation of latitude and longitude using the Haversine equation is a fundamental tool in the field of geolocation and navigation. The Haversine equation is a mathematical formula that allows for estimating the distance between two points on the Earth’s surface, considering their location in terms of latitude and longitude coordinates. This equation takes into account the curvature of the Earth and considers the average radius of the planet to calculate the distance on a sphere. Equation (1) calculates the distance between two geolocations [19]:

(1) $$D_{r}=\sqrt{{\left(2r\cdot arcsin\left(\sqrt{sin^{2}\left(\frac{\varphi_{2}-\varphi_{1}}{2}\right)+cos\varphi_{1}cos\varphi_{2}sin^{2}\left(\frac{\lambda_{2}-\lambda_{1}}{2}\right)}\right)\right)}^{2}+h^{2}}$$

where $D_{r}$ represents the distance between Tx-Rx, r is the average radius of the Earth (e.g., approximately 6371 km); $\varphi_{1}$ and $\lambda_{1}$ are the latitude and longitude coordinates of the first point, respectively; $\varphi_{2}$ and $\lambda_{2}$ are the latitude and longitude coordinates of the second point, respectively; *h* is the height difference between Tx and Rx relative to the ground.

#### 3.2.2. Propagation Loss Calculation

The expected signal power (ESP) according to [37] is defined as the signal power at the receiver Equation (2):

(2) $$ESP_{\left(dB\right)}=RSSI_{\left(dBm\right)}+SNR_{\left(dB\right)}-10\cdot log_{10}^{\left(1+10\frac{SNR_{\left(dB\right)}}{10}\right)}$$

Thus, we can calculate the propagation loss using the link balance Equation (3), as follows:

(3) $$PL_{\left(dB\right)}=P_{Tx(dBm)}+G_{\left(dBi\right)}-ESP_{\left(dB\right)}$$

ESP is the expected signal power at a certain point in dB; $P_{Tx}$ is the power transmitted by the gateway in dBm; *G* is the sum of the transmission and reception gains, usually in dBi, and finally, PL represents the propagation loss of the signal in dB.

## 4. Empirical Propagation Models

In this section, we will describe the empirical models from the literature used in this work for comparison with the ML models.

### 4.1. Floating–Intercept Model (FI)

The floating–intercept (FI) propagation model, also known as the alpha–beta (AB) model, does not have parameters based on physical fundamentals. It utilizes curve-fitting factors calculated by the minimum mean square error (MMSE) method. The model has been adopted by the 3rd Generation Partnership Project (3GPP) and the WINNER II association, which provide standard propagation models. The 3GPP and WINNER II models are widely used in the industry as they cover various types of scenarios. However, they are only used for frequencies below 6 GHz and need to be enhanced for higher frequency bands [38].

For this reason, the FI model has been extensively studied. It can be used to characterize millimeter-wave frequencies, where channels may be in line-of-sight or non-line-of-sight environments [38,39]. The equation of the FI model is given in (4):

(4) $$PL_{FI(dB)}=\alpha +10\beta log_{10}\left(\frac{d}{d_{0}}\right)-X_{\sigma FI}$$

In which α is the floating intercept coefficient in dB, known as the offset, β is the slope of the line, d is the distance between the antennas in meters, and $X_{\sigma FI}$ is the slow fading variable, which describes large-scale signal fluctuations over the average path loss, with zero mean and standard deviation σ. The values of α and β are calculated by MMSE to minimize the value of $X_{\sigma FI}$ [40]. A dataset obtained from measurements is necessary to compute the path loss values. The coefficients are then obtained from these values using established formulas [38] that minimize the standard deviation.

The FI model can be further expanded into another model known as ABG (alpha–beta–gamma), which adds another fitting parameter for frequency. Like the FI model, it has no physical anchoring and provides a curve that best fits the dataset across multiple frequencies simultaneously.

### 4.2. Close-in Model (CI)

This path loss model is referenced as the Free Space Path Loss (FSPL) provided in the equation and parameterized by the model parameter “*n*”, also known as the Path Loss Exponent (PLE) [41]. In this model, the PLE is modeled through MMSE [40], aiming to minimize the standard deviation (σ) between the Path Loss and the measured data. This model has been used for frequencies on the order of gigahertz (GHz), and it is based on the fundamental principles of propagation, linked to the Friis and Bullington formulas related to Free Space Path Loss (FSPL) [38]. While the FI model uses constants based on curve fitting, the CI model has the FSPL as a physical anchor, ensuring a fixed and continuous relationship between transmitted power and distance. Its equation is given in (5):

(5) $$PL^{CI}\left(f,d\right)=FSPL\left(f,d_{0}\right)+10nlog_{10}\left(\frac{d}{d_{0}}\right)+X_{\sigma }^{CI}$$

In which *d* is the distance between the antennas in meters, *n* is the path loss exponent, which typically has values less than 2 in indoor environments with line-of-sight, and $X_{\sigma }$ represents large-scale shadowing. The FSPL(*f*,$d_{0}$) is defined as follows:

(6) $$FSPL\left(f,d\right)=10nlog_{10}{\left(\frac{4\pi d_{0}}{\lambda }\right)}^{2}$$

where λ is the wavelength, and the distance $d_{0}$ is equal to 1 m, as it is a reference adopted in various models capable of providing a standardized modeling approach.

### 4.3. Empirical Models for Vegetation

The article [42,43] shows the most commonly used propagation models in vegetated environments for planning and deploying WSNs. These models exhibit high efficiency in path loss estimation, along with low mathematical complexity. They are known as the “Modified Exponential Decay Model (MED)” due to their format:

(7) $$Att_{MED}=xf^{y}d^{z}$$

where $Att_{MED}$ is the attenuation in dB added by vegetation on top of the Free Space Path Loss (FSPL), *f* is the frequency in GHz for the Weissberger model and in MHz for the other models, *d* is the depth distance into the vegetation in meters, and *x*, *y*, and *z* are parameters that should be adjusted based on measurements taken in each scenario where their use is required, as stated in [38].

#### 4.3.1. Weissberger Model

This model is applicable in situations where wireless signal propagation occurs in wooded environments, and the distance between the transmitting and receiving antennas must be up to 400 m, in the frequency range from 230 MHz to 96 GHz [44]. Its equation for calculating excess loss, added to the free space loss, is given by:

(8) $$Att_{WEIS}=\left\{\begin{array}{cc} 1.33\times f^{0.284}\times d^{0.588},se & 14\;m<d<400\;m\\ 0.45\times f^{0.284}\times d,se & 0\;m<d<14\;m \end{array}\right.$$

where

- $Att_{WEIS}$ is the excess attenuation according to the Weissberger model (dB);
- *d* is the distance between Tx and Rx (m);
- *f* is the system’s operating frequency (MHz).

#### 4.3.2. ITU-R Model

This model became known as the Early ITU model, proposed by the International Telecommunication Union (ITU) based on measurement campaigns in 1988. It is valid for frequencies between 200 MHz and 95 GHz and for distances between the transmitting and receiving antennas of less than 400 m [45]. Its equation for calculating excess loss, added to the free space loss, is given by (9):

(9) $$Att_{ITU-R}=0.2\times f^{0.3}\times d^{0.6},\;\;\;d<400\;m$$

- $Att_{ITU-R}$ is the excess attenuation according to the ITU-R model (dB);
- *d* is the distance between Tx and Rx (m);
- *f* is the system’s operating frequency (MHz).

### 4.4. COST 235 Model

This model distinguishes between wooded environments with the presence of leaves and those without. In this study, the equation with the presence of leaves is considered [44]. The equations for calculating excess loss added to the free space loss are given by:

(10) $$Att_{COST}=\left\{\begin{array}{cc} 26.6\times f^{-0.2}\times d^{0.5},se & semfolhas\\ 15.6\times f^{-0.009}\times d^{0.26},se & comfolhas \end{array}\right.$$

- $Att_{COST}$ is the excess attenuation according to the COST235 model (dB);
- *d* is the distance between Tx and Rx (m);
- *f* is the system’s operating frequency (MHz).

### 4.5. Fitted ITU-R (FITU-R) Model

This model, originated as an enhancement of the ITU-R model, introduced the concept of differentiating prediction model equations based on the seasonality experienced by vegetation [44]. In this study, the equation with the presence of leaves is considered. The equations for calculating excess loss added to the free space loss are as follows:

(11) $$Att_{FITUR}=\left\{\begin{array}{cc} 0.37\times f^{-0.18}\times d^{0.59},se & semfolhas\\ 0.39\times f^{-0.39}\times d^{0.25},se & comfolhas \end{array}\right.$$

- $Att_{FITUR}$ is the excess attenuation according to the FITU-R model (dB);
- *d* is the distance between Tx and Rx (m);
- *f* is the system’s operating frequency (MHz).

## 5. Machine Learning (ML) Techniques

According to [46], ML techniques can be classified into supervised and unsupervised. Supervised techniques are the most commonly used and are associated with data pairs (x, y), where x is the input to the ML model mapped to a specific output y, unlike unsupervised techniques where only the input x is known. In this research, two supervised ML techniques, MLPs and GRNNs, were used.

### 5.1. Multilayer Perceptrons (MLPs)

MLPs are an artificial neural network architecture composed of multiple layers of neurons, which are basic processing units. These networks are designed to solve complex classification and regression problems and are known for their ability to handle nonlinear data [47].

The main characteristic of MLPs is the presence of one or more hidden layers, situated between the input layer and the output layer. Each layer consists of interconnected neurons, where the connections between neurons have associated weights. These weights are adjusted during the network training process to optimize performance and enhance accuracy [47].

MLPs are capable of learning from examples provided during training. Data propagation occurs directly from the input layer to the output layer through a weighted linear combination of input values and synaptic weights. Subsequently, the output undergoes a nonlinear activation function, introducing nonlinearity into the network and enabling it to learn complex relationships among the data.

During the training process, synaptic weights are adjusted using learning algorithms such as back-propagation, which calculate the error between the network outputs and expected values, and iteratively update the weights to minimize this error. Once the network has been trained, it can be used to make predictions or classify new input examples. Through the process of forward propagation, the network performs a series of mathematical operations and neuron activations, processing the input data and generating a final output [47].

MLPs are widely used in areas such as pattern recognition, natural language processing, computer vision, and many other applications involving analysis and learning from complex data. Their ability to learn and generalize from examples makes them powerful models for classification and regression problems.

**Figure 5:**
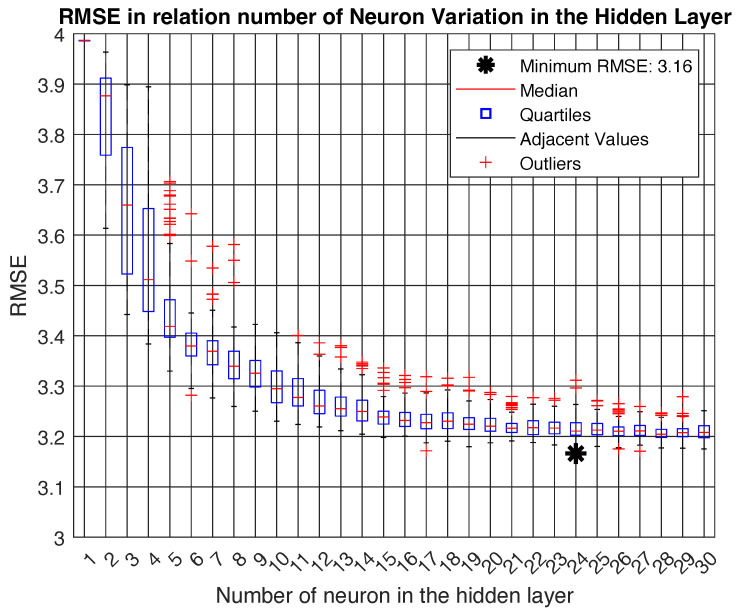
RMSE in relation to the number of neurons in the hidden layer.

Many tests were conducted considering different ANN topologies to determine the best network. The test considered neuron numbers from 1 to 30 in the hidden layer, and for each neuron in the hidden layer the test was repeated for 100 different random seeds. Finally, the topology with one hidden layer with 24 neurons yielded the best accuracy, as seen in Figure 5.

**Figure 6:**
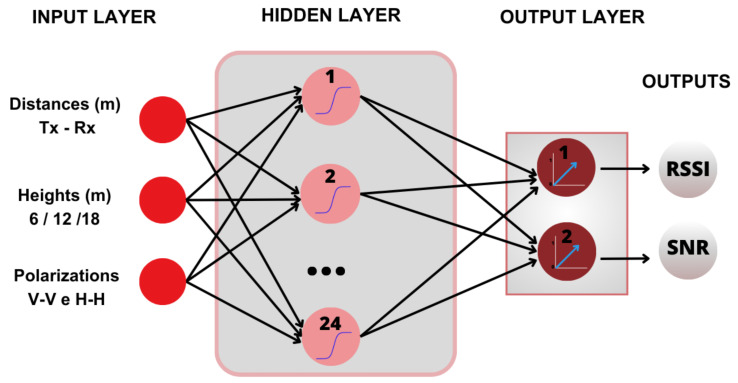
MLP network implemented.

Figure 6 represents the MLP neural network modeled for this research, in which the input layer has three neurons, which represent the distance between the transmitter (Tx) and the receiver (Rx) in meters (m), the heights of the Tx, 6, 12, and 18, in meters (m) and the co-polarizations of the transmitting and receiving antennas (VV and HH), with sigmoid function in the hidden layer and linear activation function for the output layer.

### 5.2. General Regression Neural Network (GRNN)

The general regression neural network (GRNN) is a type of artificial neural network developed to solve regression problems, i.e., predict continuous values in response to a set of input variables. The GRNN was originally proposed by Donald F. Specht in 1991 as an extension of the probabilistic classification algorithm called probabilistic neural network (PNN) [48].

GRNN is a feedforward neural network that stands out for its simplicity and ease of implementation. It is a variant of the RBF network, which uses radial basis functions to perform interpolation and extrapolation of data and consists of four main layers: the input layer, the pattern layer, the summation layer, and the output layer [48].

In the input layer, the input variables are normalized and provided as input to the network. Then, in the pattern layer, the network compares the input patterns with stored training patterns. These training patterns consist of the input and corresponding output values’ combination. Each training pattern has an associated activation function that measures the similarity between the input pattern and the training pattern. Typically, the Gaussian activation function is used where the output is a measure of the distance between the input pattern and the training pattern.

The summation layer receives the activation values calculated in the pattern layer and performs a weighted sum of these values. The weights associated with the activation values are determined by the Gaussian activation function, which assigns a higher weight to training patterns closer to the input pattern.

Finally, the output layer combines the outputs from the summation layer to generate a final response, which is the value predicted by the GRNN. The output is calculated by the patterns layer’s output values’ weighted average, where the weights are determined by the Gaussian activation function.

One of the advantages of GRNN is its ability for fast and efficient learning. Once the network is trained with corresponding input and output patterns, it can rapidly predict output values for new input patterns without the need for an iterative process of weight adjustment [48].

Additionally, the GRNN is known for being a robust neural network capable of handling noise and incomplete data. It also has a low number of hyper-parameters. In this case, it had only one, the smooth parameter, which for this model was set to 0.0039, making it easier to implement and adjust.

However, it is important to note that the GRNN may be more suitable for simple regression problems with a limited number of input variables. For more complex problems or large datasets, other neural network architectures such as deep neural networks may be more appropriate.

**Figure 7:**
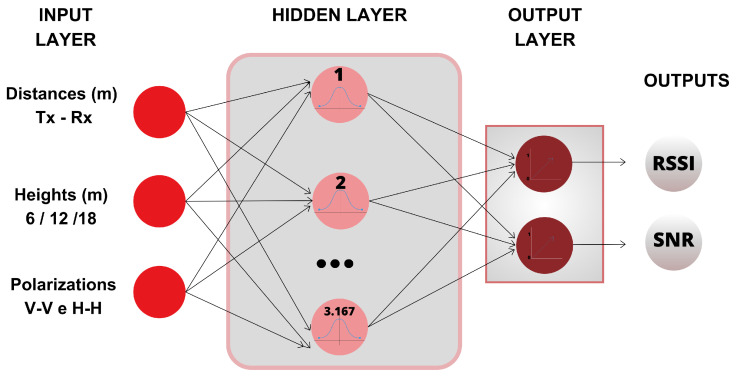
GRNN network implemented.

Figure 7 represents the architecture of the GRNN used in this research, considering the same inputs as the MLP and also predicting RSSI and SNR as output.

### 5.3. Dataset

The dataset used for implementing both techniques, MLP and GRNN, consists of 4525 samples distributed across three randomly generated datasets: the training set containing 3167 points, which corresponds to 70% of the total data; and the validation set and the test set, each containing 679 points, amounting to 30% of the total samples, as shown in Table 3.

The training process involves presenting a pattern to the input layer units, where the units compute their response and present it to the output layer to obtain the network answer. Then, the error is computed and propagated from the output layer back to the input layer, and the weights of the connections in hidden layer units are adjusted, gradually decreasing the error to achieve the best generalization rate of the MLP. In the supervised training of the MLP, the Levenberg–Marquardt algorithm was used.

## 6. Results

In this section, the results obtained in the typical Amazonian forest, where the vegetation has an average height of 24 m, and the transmitter heights are defined at 6 m (tree trunk), 12 m (beginning of the tree canopy), and 18 m (mid-canopy), will be presented. The influence of these antenna’s heights and polarizations in signal propagation will be analyzed; following this, comparisons will be made between the proposed RNA-based models with other propagation loss models such as CI, FI, Weissberger, Early ITU-R, Cost235, and FITU-R models, aiming to demonstrate the efficiency of using ML techniques such as MLP and GRNN in estimating propagation loss in wooded environments.

### 6.1. Analysis of the Influence of Heights and Polarizations in Densely Wooded Environments

Wireless communication in densely vegetated environments has been gaining increasing importance due to applications such as environmental monitoring, fire prevention, and search and rescue systems. However, dense vegetation, especially tree canopies, can cause signal attenuation and interference, making it essential to properly select the polarization and height of the transmitter to improve the reliability and quality of transmissions.

Wireless signal transmissions in such environments pose unique challenges, where the proper choice of electromagnetic wave polarization can significantly affect transmission performance in this scenario. This section explores the effectiveness of HH (horizontal transmitted and horizontal received) and VV (vertical transmitted and vertical received) polarizations at different heights relative to transmissions at both trunk and canopy levels of trees.

The choice of microwave polarization (such as HH and VV) at different heights of transmissions below tree canopies is related to the interaction of electromagnetic waves with vegetation. This interaction can vary depending on the structure and density of the vegetation, as well as the characteristics of the waves used.

The relationship between RSSI and SNR in LoRa technology is that RSSI provides information about the overall signal strength, while SNR complements this information by indicating the signal quality relative to background noise. To obtain a more comprehensive and accurate view of the LoRa communication link quality, it is important to consider both parameters. In some LoRa implementations, SNR may be more significant in determining data reception capability than RSSI, especially in environments with high noise or interference.

**Figure 8:**
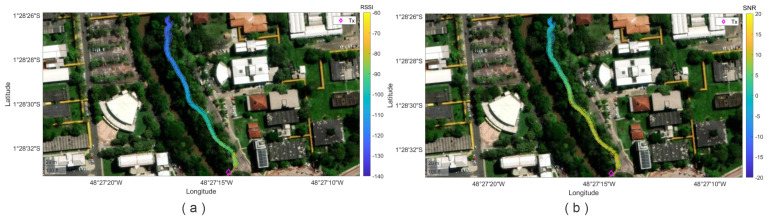
(**a**) The collected GPS data corresponding to RSSI levels vs. distance in the studied environment. (**b**) The collected GPS data corresponding to SNR levels vs. distance in the studied environment.

Figure 8 illustrates the measurement points which are in the middle of a dense forest. The transmitter is at the beginning of the way and has a static longitude and latitude; in contrast, the receiver varies in longitude and latitude since it is moving across the way. Figure 8a,b show the RSSI and SNR values, respectively. A detailed analysis of RSSI and SNR according to the transmitter height is below.

**Figure 9:**
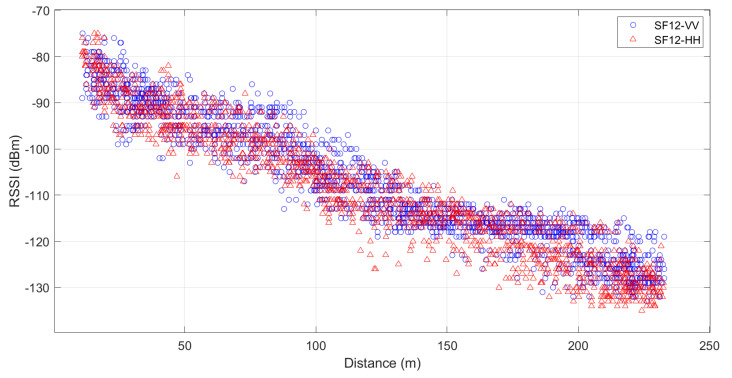
Distribution of the measured RSSI data with respect to distance for SF12 at all heights for HH and VV polarizations.

According with Figure 9, the lowest value for RSSI with vertical polarization is −133 dBm and the highest value is −75 dBm; on the other hand, for horizontal polarization, the lowest value is −135 dBm and the highest value is −75 dBm. In addition, it can be seen that the RSSI values are similar for both polarizations; however, RSSI values for VV show higher values, especially for distances greater than 150 m.

Additionally, four VV RSSI values show a decreasing trend around 20 dB each 100 m. For HH, the decrease in RSSI values is around 25 dB each 100 m. This means that the forest plays a significant attenuation role, even over short distances.

**Figure 10:**
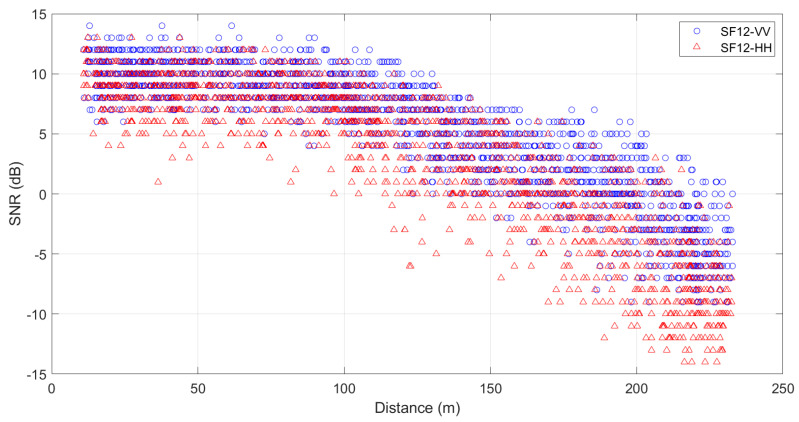
Distribution of the measured SNR data with respect to distance for SF12 at all heights for HH and VV polarizations.

Figure 10 shows that the minimum SNR for HH is −13, and for VV is −9, the maximum value for HH is 13, and for VV it is 14. Also, the SNR for both polarizations begins decreasing for distances greater than 100 m for SF12 at all heights. However, VV polarization has a stronger and better signal quality in a densely wooded environment, which means that the signal RSSI is stronger relative to the noise, resulting in better communication performance. Additionally, when observing the variations in RSSI and SNR values with distance, it is noted that the forest environment affects both RSSI and SNR even at short distances.

In summary, according to Figure 9 and Figure 10, it can be observed that VV polarization performs better in transmission compared to HH polarization for all heights. Furthermore, in all these situations, it is observed that RSSI exhibited a linear behavior, and SNR exhibited an exponential behavior for a distance over 250 m.

#### Analysis of RSSI and SNR Values with Respect to Heights

The results of RSSI and SNR for SF12 in densely wooded environments for IoT networks in the 915 MHz frequency band are presented and analyzed for each height. A new graph that condenses the information of RSSI, mean, and standard deviation values for fixed distance intervals is presented to make it easy to compare for different heights and polarizations. The mean and standard deviation are calculated considering all RSSI values contained within the distance interval covered by a sliding window.

**Figure 11:**
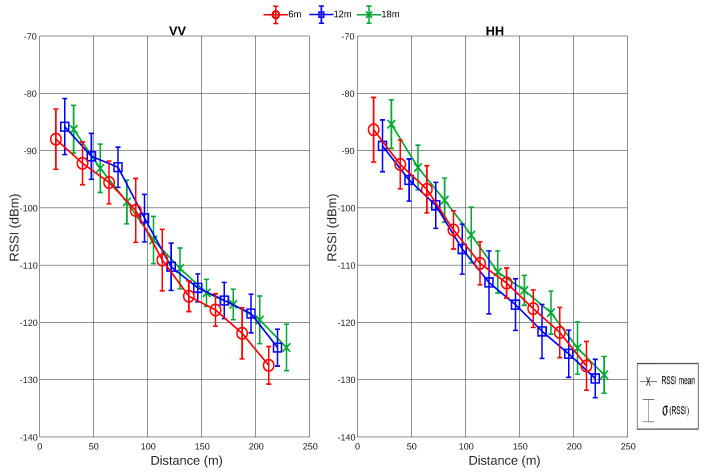
Analysis of the mean and standard deviation values of RSSI for SF12 at all heights and for VV and HH polarizations.

**Figure 12:**
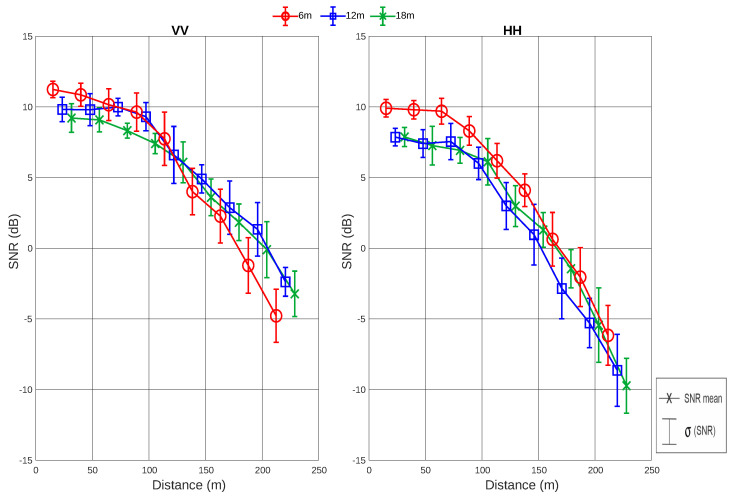
Analysis of the mean and standard deviation values of SNR for SF12 at all heights and for VV and HH polarizations.

The RSSI and SNR values along the traveled path are displayed for the SF12 combination heights of 6, 12, and 18 m in Figure 11 and Figure 12. First, while analyzing the horizontal antenna polarization (HH), for a height of 6 m, where the transmitter is located at the tree trunk level, it can observed that the RSSI values range from −131 to −80 dBm, while the SNR ranges from −8 to 11 dB.

At a height of 12 m, corresponding to the beginning of the tree canopy, the RSSI values range from −133 to −85 dBm, while the SNR ranges from −11 to 8 dB. Finally, at a height of 18 m, corresponding to the middle of the tree canopy, the RSSI values range from −132 to −82 dBm, while the SNR ranges from −12 to 8 dB.

Analyzing the vertical antenna polarization (VV), the RSSI and SNR values at a height of 6 m range from −131 to −83 dBm and from −7 to 12 dB, respectively. At a height of 12 m, the RSSI values are in a range from −128 to −81 dBm, and the SNR values are in a range from −3 to 11 dB. Finally, for a height of 18 m, the RSSI values are in a range from −129 to −82 dBm, and the SNR values are in a range from −5 to 10 dB.

From Figure 11 and Figure 12, it is possible to observe that the VV polarization presents RSSI and SNR values similar to the HH polarization. However, the decay in the VV signal level over the distance is smaller compared to the HH signal. In addition, analyzing Table 4, it can also be verified that, on average, the lowest value of VV polarization (at a height of 18 m) shows a result practically equal to the highest value of HH polarization (at a height of 6 m).

In summary, when placing the transmitter at heights of 12 and 18 m, corresponding to the beginning and middle of the leaves, respectively, a worse SNR is observed. Therefore, what most affects the signal quality in a wooded environment are the leaves. Furthermore, analyzing the mean values of RSSI and SNR for each situation, it is observed that, as the heights increase, the mean values of both RSSI and SNR decrease, as shown in Table 4.

### 6.2. Adjustments to CI and FI Propagation Models

The values of PLE and the coefficients alpha (α) and beta (β) are crucial for improving the accuracy of signal propagation predictions in real environments, which enhances the performance of wireless networks in vegetated areas. All values of PLE for the CI model and the coefficients (α and β) and initial distance ($d_{0}$) for the FI model are displayed in Table 5.

According to the values of FI(β) and CI (PLE), at a height of 6 m (tree trunk), the propagation loss for both VV and HH polarizations is similar. However, at heights of 12 m (beginning of the tree canopy) and 18 m (middle of the tree canopy), the VV polarization has the lowest decay with distance.

### 6.3. Evaluation of the Proposed Propagation Models Based on Artificial Neural Networks (ANNs)

The proposed models use three input variables: distance (5 to 250 m), transmitter height (6, 12, and 18 m), and the co-polarization of the transmitting and receiving antennas (HH-VV). To evaluate the accuracy and precision of the proposed models, two metrics were applied: root mean square error (RMSE) and standard deviation (σ).

RMSE measures the square root of the squared errors’ average between the values predicted by the model and the measured values. Its goal is to predict a continuous numerical value, such as propagation loss in a wireless communication system. The RMSE is calculated using Equation (12) [49]:

(12) $$RMSE=\sqrt{\frac{1}{n}\sum_{i=1}^{n}{\left(y_{i}-{\hat{y}}_{i}\right)}^{2}}$$

in which ∑ indicates the sum of all elements for each sample *i*, $y_{i}$ represents the actual observed value in sample *i*, ${\hat{y}}_{i}$ represents the value predicted by the model for the i−th sample, and *n* is the total number of samples or observations in the dataset.

The σ is a measure of dispersion or variability in the data relative to the mean. In simple terms, it tells us how far the values are from the mean. The standard deviation is calculated using the Formula (13) [50]:

(13) $$\sigma =\sqrt{\frac{\sum_{i=1}^{n}{\left(x_{i}-\bar{x}\right)}^{2}}{n}}$$

in which ∑ indicates the sum of all elements, $x_{i}$ is the individual value in the dataset, $\bar{x}$ is the mean of the values, and *n* is the total number of examples.

The importance of standard deviation in the evaluation of RNA-based propagation loss models is related to its ability to show us the variability of observed propagation loss values. The smaller the standard deviation, the more consistent and accurate the results predicted by the model.

To properly evaluate an RNA-based propagation loss model, it is essential to consider both RMSE and standard deviation. RMSE provides a general idea of the model’s predictions accuracy, while the standard deviation helps to understand the variability in the data and the prediction’s reliability across different scenarios.

Furthermore, it is important to emphasize that the choice of evaluation metrics also depends on the context and specific requirements of the problem at hand. For example, in critical applications such as communication systems, it is essential to have propagation loss prediction models with high accuracy and low standard deviation to ensure the proper performance of the system.

**Figure 13:**
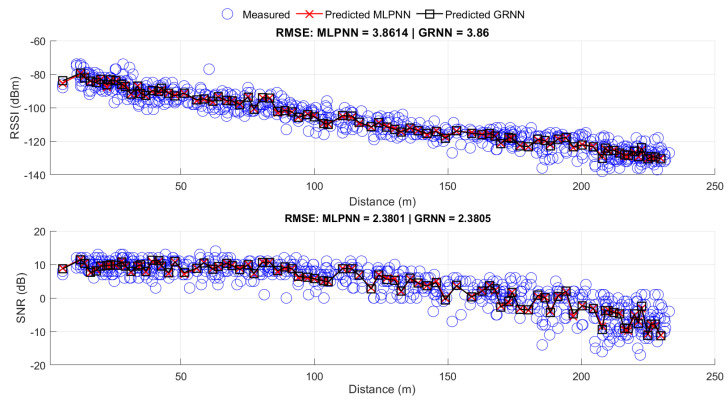
Measured data and prediction of MLPNN and GRNN.

The obtained results were close in terms of performance, as shown in Figure 13, and the MLP responses, represented by the red x, are close to GRNN responses, represented by the black square. Moreover, the RMSE of 3.86 for the GRNN and 3.8614 for the MLPNN suggests that both networks were able to effectively capture patterns in the data and generalize to new samples. This closeness in results may also indicate that the problem is relatively well-behaved and that both networks are providing consistent and accurate solutions.

Table 6 shows the values of the evaluated metrics. It can be observed that both ANNs were very close in terms of accuracy and precision. However, regarding training time, the GRNN was approximately 82% faster than the MLPNN. Both were trained using a notebook equipped with a Core i5 12500H processor. The advantage of the GRNN in requiring lower computational cost is an important characteristic to consider, especially when addressing large volumes of data or when optimizing available resources.

### 6.4. Comparison with Propagation Loss Models from the Literature

The results presented by the proposed RNA-based models for densely forested environments for IoT networks in the 915 MHz frequency range were compared with empirical models CI and FI, as well as with models adapted for vegetated environments, namely Weissberger, Early ITU-R, Cost235, and FITU-R.

**Figure 14:**
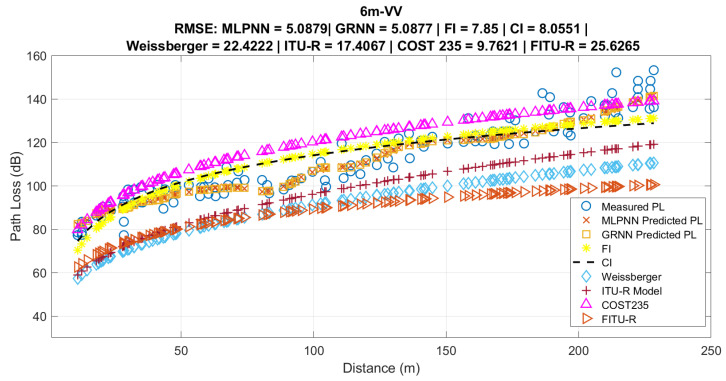
Propagation loss models and applied and proposed MLPNN and GRNN models.

Figure 14 shows the comparison of the proposed RNA-based models and vegetation-adapted models to the measured data for a height of 6 m VV. As seen in Figure 13, the MLPNN and GRNN models better represented the average behavior of the measured data, obtaining RMSE and standard deviation values that were similar and lower than the other analyzed models. This is due to RNA-based propagation models being able to estimate the variability in propagation loss more accurately at a single point, as well as representing the signal behavior in the studied environment.

The other empirical propagation models obtained higher RMSE and standard deviation values than the proposed models. In particular, the FI and CI models, which were adjusted according to the measured data using the linear least squares technique, showed higher RMSE values. However, the FI model performed slightly better than the CI model, as it considers the measured initial point as its own reference, while the CI model considers the value of FSPL as the initial reference point.

For the propagation loss models in vegetated environments, the one that best represented the measured data was COST235, followed by ITU-R, Weissberger, and finally the FITU-R model. The FITU-R model performs better than the ITU-R and Weissberger models up to the first 50 m. The comparisons of propagation models, through the RMSE and σ metrics, for the other heights and polarizations, are presented in Table 7.

## 7. Conclusions

This work aimed to develop two propagation models using machine learning techniques, MLPNN and GRNN, for densely forested environments at a frequency of 915 MHz, and to evaluate them in relation to measured data and existing models in the literature. To achieve this, an extensive measurement campaign was conducted in the Camillo Vianna forest, located within the Federal University of Pará, which features various tree species, predominantly the Pau-Mulato type.

In this measurement campaign, data related to geolocation, RSSI, and SNR were collected along a 250 m path. Subsequently, there was a need for data processing, such as calculating the distance using geolocations and calculating propagation loss using RSSI and SNR data.

Furthermore, artificial neural networks (MLPNN and GRNN) were trained, in addition to the application of least squares technique to adjust the CI and FI propagation models. The objective was to predict the average behavior of signal propagation and evaluate them in relation to the measured data. Subsequently, they were compared with models adapted for vegetation (ITU-R, Weissberger, COST235, and FITU-R).

Thus, the proposed propagation models, MLPNN and GRNN, achieved better accuracy and precision, with RMSE values of 3.8614 and 3.86, and standard deviation values of 3.8564 and 3.8558, respectively, for RSSI. In addition, for the SNR prediction, the RMSE values were 2.3801 and 2.3805, and the standard deviation values were 2.3788 and 2.3798, respectively.

The results show the models’ ability to learn from data and capture nonlinear relationships. This ability allows these models to achieve greater predictive capability of the average signal behavior compared to classical models in the literature.

The satisfactory results obtained by the MLPNN and GRNN ANNs in modeling propagation loss indicate that both are viable options for this regression task. With minimal difference in the RMSE results, the choice between the two can be guided by training speed and resource demand, with the GRNN having an advantage in these aspects.

While the CI and FI models performed overall better than vegetation-adapted propagation models, it is worth noting that both were adjusted with measured data from the environment, as shown in Table 5. As for models adjusted for vegetated environments, the COST235 propagation model generally better fitted the measured data than other vegetation models.

Another significant contribution of the work was the analysis regarding heights and co-polarizations. The appropriate choice of electromagnetic wave polarization for wireless signal transmissions in vegetated environments is crucial to optimize the efficiency and reliability of communications. This research demonstrated that VV polarization is more suitable for all heights. Understanding these differences allows for the design of more efficient and resilient communication networks, contributing to advancements in monitoring and research applications in densely forested environments. Finally, another contribution was calculating the values of PLE, α, and β for the study environment, as shown in Table 5.

For future work, the intention is to analyze the impact of spreading factor, and consequently, the bit rate, as the two are directly related. Also, analyze the heights higher than 18 m and lower than 6 m to quantify the impact of dense vegetation on electromagnetic signals. To examine the cross-polarizations of transmitter and receiver, antennas for different heights need to be used in order to understand which polarization type experiences less loss through the forest. Additionally, due the propagation loss imposed by vegetation on transmission across these various scenarios, the intention is to utilize other machine learning techniques (such as ANFIS) and statistical metrics (including MAE, MPE, MAPE, and GRG-MAPE), as well as employ the ITU-R P.1546-6 model as it is the new extension of the 1546 model for distances shorter than 1000 km.

## Figures and Tables

**Table 1:** Configuration of the measurement setup parameters.

Operating Frequency	Effective Radiated Power	Spreading Factor	Bandwidth	Coding Rate
915 MHz	5 dBm	12	125 KHz	4/5

**Table 2:** Configuration corresponding to the measurement methodology.

SF	Heights	Antenna Polarization
12	6 m 12 m 18 m	VV HH

**Table 3:** Splitting the dataset.

Sample Set	Number of Samples	% in Relation to the Total
Training Validation Test	3167 679 679	70% 15% 15%

**Table 4:** Statistical metrics of the collected signal.

Height	σ-RSSI (dBm)		Mean RSSI (dBm)		σ-SNR (dB)		Mean SNR (dB)	
	HH	VV	HH	VV	HH	VV	HH	VV
6 m	14.84	16.62	−107.24	−105.76	6.68	6.93	4.32	5.36
12 m	14.86	15.01	−111.09	−106.24	6.87	5.13	1.16	5.10
18 m	15.91	14.90	−108.76	−107.31	7.22	5.96	1.08	4.28

**Table 5:** Adjusted coefficient values for FI and CI.

SITUATIONS	FI ($d_{0}$ (m)|α|β)	CI (PLE)
SF12-6 m-HH SF12-6 m-VV SF12-12 m-HH SF12-12 m-VV SF12-18 m-HH SF12-18 m-VV	10.92|69.02|4.98 10.92|68.29|4.91 17.32|76.56|5.87 12.45|68.34|4.79 14.13|71.43|5.66 12.47|73.30|4.71	3.93 3.85 4.15 3.73 4.08 3.86

**Table 6:** Evaluation metrics for the ANNs.

ANNs	RMSE	σ	Training Time (s)
GRNN MLPNN	3.8600 3.8614	3.8558 3.8564	0.3634 2.0839

**Table 7:** Results of the propagation models’ comparison.

Type	Metrics	GRNN	MLPNN	CI	FI	WEISSB.	ITU-R	COST 235	FITU-R
SF12-6 m-HH	RMSE	3.46	3.39	7.83	7.69	22.12	16.90	9.91	25.70
	σ	4.21	4.14	7.86	7.73	6.51	6.17	17.23	9.30
SF12-6 m-VV	RMSE	4.12	4.11	8.05	7.85	22.42	17.40	9.76	25.62
	σ	4.97	4.96	8.08	7.88	6.95	6.46	18.73	9.95
SF12-12 m-HH	RMSE	4.11	4.19	8.11	7.84	26.70	21.30	7.83	30.40
	σ	5.71	5.80	8.15	7.89	7.35	6.73	17.77	10.21
SF12-12 m-VV	RMSE	3.66	3.61	7.44	7.42	19.59	14.50	11.75	23.32
	σ	4.29	4.24	7.45	7.45	6.09	6.35	16.00	8.16
SF12-18 m-HH	RMSE	4.01	4.04	9.38	8.53	23.99	18.43	10.01	28.26
	σ	5.30	5.31	9.41	8.58	8.20	7.30	19.13	11.48
SF12-18 m-VV	RMSE	3.82	3.86	7.24	7.10	21.42	16.21	10.10	25.10
	σ	5.11	5.16	7.27	7.13	6.22	6.12	16.72	8.77

## Data Availability

The authors reserve the right not to disclose the private dataset used in this study.

## Abbreviations

The following abbreviations are used in this manuscript:

UFPA	Federal University of Pará
LOS	Line-of-sight
NLOS	Non-line-of-sight
IOT	Internet of Things
LoRA	Long Range
MMSE	Minimum mean square error
RMSE	Root mean squared error
WSNs	Wireless sensor
5G	Fifth generation
ML	Machine learning
RNAs	Artificial neural networks
SF	Spreading factor
GRG-MAPE	Maximization of gray relationship degree and mean absolute percentage error
UAVs	Unmanned aerial vehicles
LoRaWan	Long range wide area network
SNR	Signal-to-noise ratio
Tx	Transmitter
Rx	Receiver
VV	Vertical–vertical
HH	Horizontal–horizontal
PLE	Path loss exponent
FI	Floating intercept
CI	Close in
RSSI	Received signal strength indicator
GRNN	General regression neural network
MLPNN	Multi-layer perceptron neural network
